# Localization of CD26/DPPIV in nucleus and its nuclear translocation enhanced by anti-CD26 monoclonal antibody with anti-tumor effect

**DOI:** 10.1186/1475-2867-9-17

**Published:** 2009-06-26

**Authors:** Kohji Yamada, Mutsumi Hayashi, Wenlin Du, Kei Ohnuma, Michiie Sakamoto, Chikao Morimoto, Taketo Yamada

**Affiliations:** 1 Department of Pathology, Keio University School of Medicine, Tokyo, Japan; 2 Division of Clinical Immunology, Advanced Clinical Research Center, Institute of Medical Science, University of Tokyo, Tokyo, Japan

## Abstract

**Background:**

CD26 is a type II, cell surface glycoprotein known as dipeptidyl peptidase (DPP) IV. Previous studies have revealed CD26 expression in T cell leukemia/lymphoma and malignant mesothelioma, and an inhibitory effect of anti-CD26 monoclonal antibody (mAb) against the growth of CD26+ cancer cells in vitro and in vivo. The function of CD26 in tumor development is unknown and the machinery with which the CD26 mAb induces its anti-tumor effect remains uncharacterized.

**Results:**

The localization of CD26 in the nucleus of T cell leukemia/lymphoma cells and mesothelioma cells was shown by biochemical and immuno-electron microscopic analysis. The DPPIV enzyme activity was revealed in the nuclear fraction of T cell leukemia/lymphoma cells. These expressions of intra-nuclear CD26 were augmented by treatment with the CD26 mAb, 1F7, with anti-tumor effect against the CD26+ T cell leukemia/lymphoma cells. In contrast, the CD26 mAb, 5F8, without anti-tumor effect, did not augment CD26 expressions in the nucleus. Biotin-labeled, cell surface CD26 translocated into the nucleus constantly, and this translocation was enhanced with 1F7 treatment but not with 5F8.

**Conclusion:**

These results indicate that the intra-nuclear CD26 which moves from plasma membrane may play certain roles in cell growth of human cancer cells.

## Background

CD26 is a 110 kDa transmembrane glycoprotein expressed in many different cells and tissues, including T lymphocytes, melanocytes, epithelia of the renal tubule or colonic mucosa, and endothelial cells. It consists of three domains, a cytoplasmic short tail domain with only 6 amine residues, a transmembrane domain, and an extracellular domain. This extracellular domain possesses the activity of a membrane-associated ectopeptidase known as dipeptidyl peptidase (DPP) IV that preferentially cleaves N-terminal dipeptides from polypeptides with L-proline or L-alanine in the penultimate position. It binds proteins such as adenosine deaminase (ADA), CD45, and a part of the extracellular matrix (ECM) [1]. On T lymphocytes CD26 regulates expression of ADA on the cell surface, with the CD26/ADA complex playing a key role in the catalytic removal of local adenosine to regulate immune system function [2]. CD26 also functions as a co-stimulatory factor in the CD3 and CD2 pathways of activation, as well as acting as an alternate pathway for T lymphocyte activation when cross-linked with solid-phase, immobilized specific monoclonal antibodies (mAbs) [3].

CD26 is expressed in many kinds of human malignancy, including mesothelioma, renal cell carcinoma, and T cell leukemia/lymphoma. CD26 expression is observed in mesothelioma cells, but not in non-neoplastic mesothelial cells [4]. The murine CD26 mAbs, 1F7 and 14D10, which recognize the cell membrane-proximal glycosylated region starting with a 20-amino acid flexible stalk region of human CD26, are reported to have anti-tumor effects against T cell leukemia/lymphoma, mesothelioma and renal carcinoma cells, both *in vitro *and *in vivo *[4-6]. These mAbs induce the arrest of the G1/S cell cycle, concomitant with blocking the adhesion of cancer cells to the ECM in mesothelioma cells and renal carcinoma cells [4,5]. The CD26 mAb, 1F7, inhibits the cell growth of T lymphocytes and modulates the expression of cell surface CD26 in T-cell clones [3,6-8]. The 1F7-induced modulation of CD26 is found to be a dynamic process following the capping and internalization of the antigen-antibody complex. 1F7 induces p21 expression via *de novo *protein synthesis, followed by G1/S cell cycle arrest. It is, however, unknown how CD26, stimulated with 1F7, induces p21 expression.

Previous reports showed the localization of CD26 on the plasma membrane, the endoplasmic reticulum (ER), and the lysosome, by ultrastructural analysis [9,10]. We herein show with biochemical and morphological analysis, localization of CD26, not only on the cell surface and in the lysosome, but also in the nucleus of human cancer cells. We also assessed whether the anti-tumor activity of the CD26 mAb, 1F7, against T-cell leukemia/lymphoma cells, correlated with the translocation of CD26 into nucleus of human tumor cells.

## Results and discussion

### The presence of CD26/DPPIV in the nucleus

**Figure 1:**
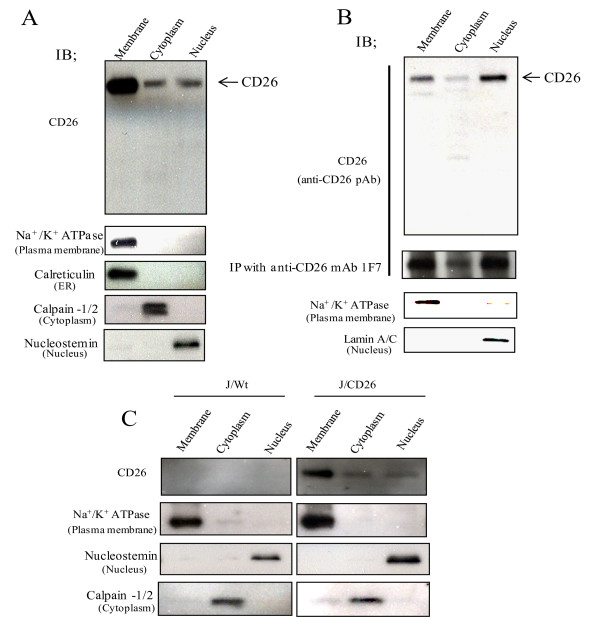
**Nuclear localization of CD26**. (A-C) Equal amounts of membrane, cytoplasm and nuclear extracts from (A) Karpas 299 cells, (B) JMN cells, and (C) J/Wt cells and J/CD26 cells were immunoblotted with CD26 pAb.

We assessed the subcellular localization of CD26 by biochemical analysis. Karpas 299 cells and JMN cells, with endogenous CD26 expression, were fractionated into membrane, cytoplasmic, or nuclear fractions. By immunoblotting, CD26 was detected as a 110 kDa band in the nuclear fraction, as well as in the membrane and cytoplasmic fractions (Fig. 1A and 1B). CD26 expression was also observed in the nuclear, membrane and cytoplasmic fractions of J/CD26 cells, but not in any fractions of J/Wt cells (known to be CD26-negative cells at both the protein and mRNA level) [11] (Fig. 1C). Mesothelioma cell line JMN relatively exhibits higher CD26 level in the nuclear fraction than that in two T cell lines, Karpas 299 and J/CD26 cells. The difference may be due to difference in cell lineages between mesothelioma cell and T lymphocyte. To confirm the 110 kDa band observed in immunoblotting was indeed CD26, CD26 was immunoprecipitated from each fraction of the JMN cells with the murine CD26 mAb, 1F7, and detected by immunoblotting with a CD26 pAb. The 110 kDa band was confirmed as the CD26 protein. However, no NLS sequence of CD26, which is defined as cluster of a series of basic amino acids, is found by PSORT II program that predicts subcellular localization . CD26 may be required to associate with other partner molecules to move to the nucleus.

**Figure 2:**
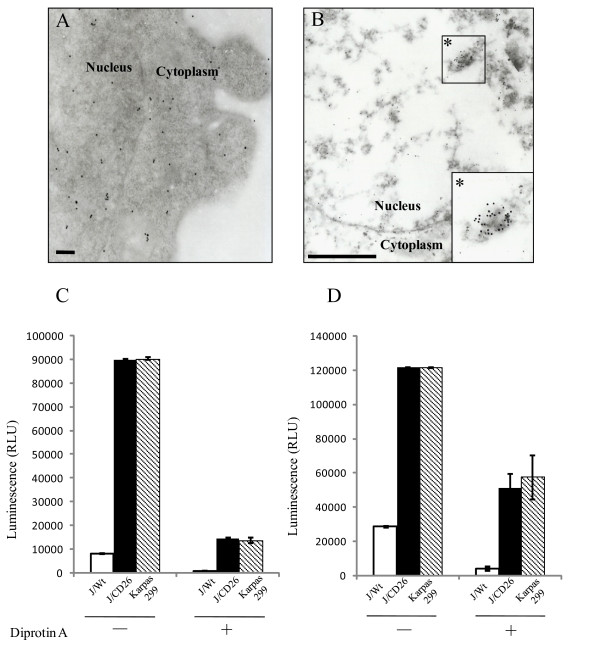
**Nuclear localization of CD26/DPPIV**. (A and B) Immuno-electron microscopy in (A) Karpas 299 cells (scale bar: 200 nm) and (B) JMN cells (scale bar: 1 μm). (C and D) The activity of DPPIV. J/Wt, J/CD26 or Karpas 299 cells were measured in (C) nuclear and (D) cytosolic fractions. Data are the means ± s.d. for each representative experiment, which was performed in triplicate.

Immunoelectron microscopic analysis of Karpas 299 and JMN cells showed immunogold labeled-CD26 on the cell surface side of the plasma membrane and in the lysosome, as previously reported [9,10] (Fig. 2A). In addition, immunogold particles were diffusely observed in the nucleus of the Karpas 299 cells (Fig. 2A), while in the JMN cells, the immunogold particles were clustered in areas of high electron density (such as the chromatin structure) in the nucleus (Fig. 2B).

The CD26 in nucleus was assessed for DPPIV enzyme activity. As a result, specific enzyme activity was detected in the nuclear fraction (and the cytosolic fraction) of both the J/CD26 cells and the Karpas 299 cells (Fig. 2C and 2D). DPPIV activity in the nuclear fraction was also observed following fractionation by an alternative method (Qproteome cell compartment kit, Qiagen) using J/CD26 cells and the JMN mesothelioma cells (data not shown). This DPPIV activity in the nuclear and cytosolic fraction was inhibited by treatment with diprotin A, an inhibitor of the DPPIV enzyme (Fig. 2C and 2D). These data suggest that CD26/DPPIV functions as a catalytic enzyme against, as yet, unidentified molecules in the nucleus.

### Effect of the CD26 mAb, 1F7, on the nuclear localization of CD26

**Figure 3:**
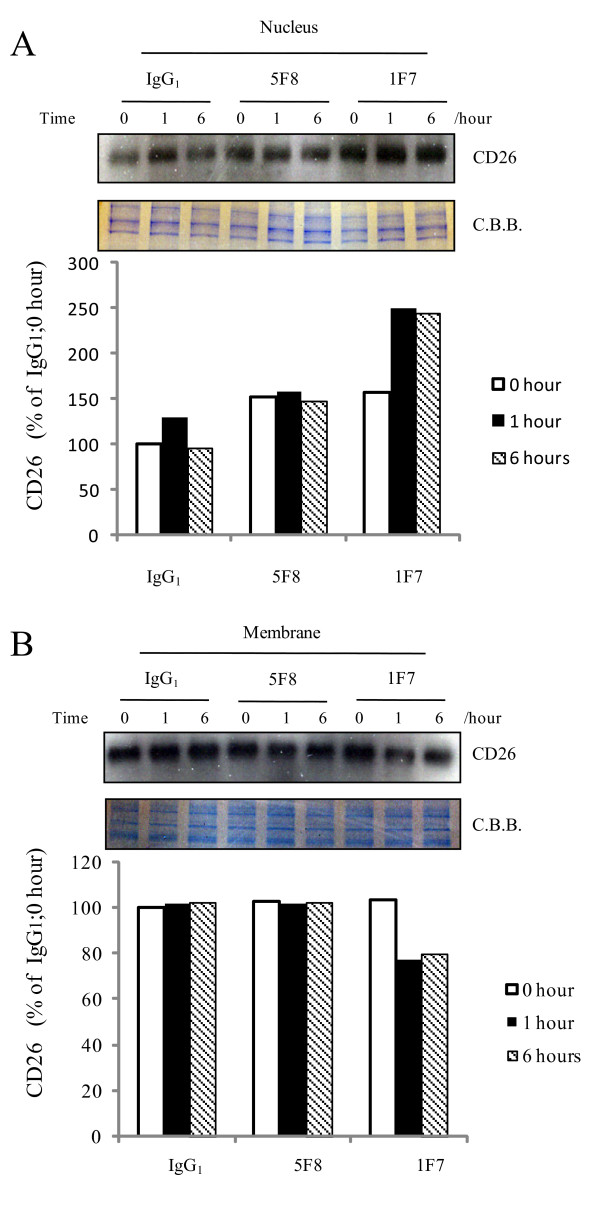
**Increase of CD26 in nucleus following treatment with CD26 mAb, 1F7**. J/CD26 cells were incubated with media containing the CD26 mAbs, 1F7 or 5F8, or control mouse IgG_1_. After incubation for 0, 1, or 6 hours, the cells were separated into (A) nuclear and (B) membrane fractions and CD26 proteins were immunoblotted. Coomassie brilliant blue (C.B.B.) staining was shown. Representative CD26 expression was shown. Similar results were obtained in three independent experiments.

We examined whether a change in CD26 localization into the nucleus could be initiated by treatment with the CD26 mAb, 1F7, since 1F7 treatment was shown to induce internalization of CD26 from the plasma membrane into the cytoplasm of CD26+ T-cells [7]. CD26 levels in the nuclear fraction of J/CD26 cells were found to be at a peak within one hour of treatment with 1F7, but not 5F8 or IgG_1 _(Fig. 3A). Accordingly, CD26 levels in the membrane fraction were decreased within one hour of 1F7 treatment (Fig. 3B). The augmentation of CD26 in the nuclear fraction following 1F7 treatment may be an extreme early event because the increased localization of CD26 in nuclear fraction was also observed at the zero hour time point. The increased amount of CD26 at zero time point seems to depend on some reactions during extra consumed time indispensable for experimental procedure. On the other hand, the CD26 mAb, 5F8, which recognizes a distinct epitope to 1F7, did not influence the nuclear localization of CD26. 1F7 reportedly inhibits the cellular proliferation of T-cells *in vitro*, while no such inhibitory effect has been observed with 5F8 [5,8]. Thus, the translocation of CD26 into the nucleus, following 1F7 treatment, may result in the inhibition of cell growth in an epitope-specific manner.

### Translocation of CD26 into the nucleus following 1F7 treatment

**Figure 4:**
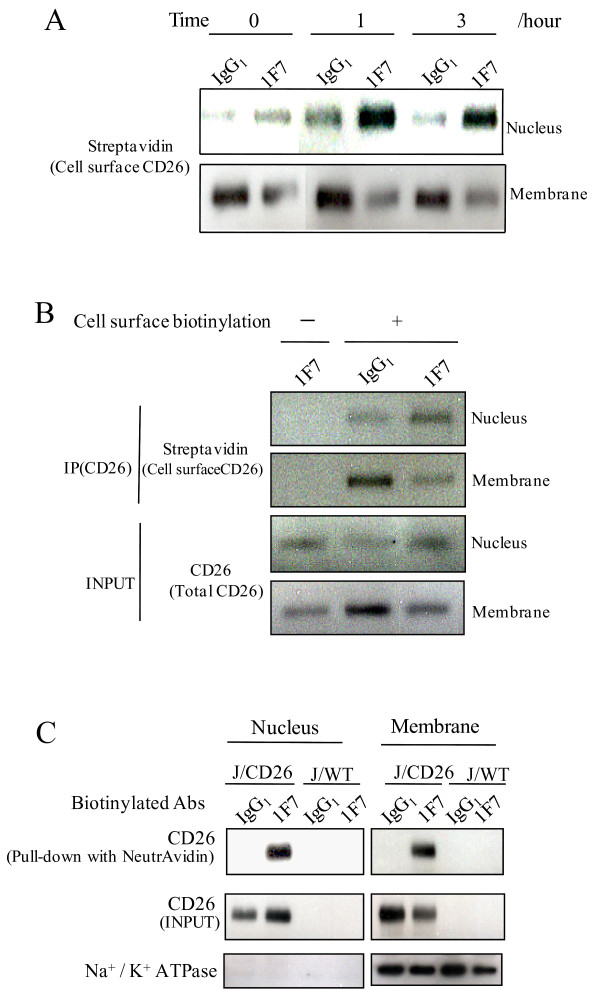
**Translocation of CD26 into nucleus from cell surface**. (A and B) Cell surface biotin-labeling of (A) J/CD26 cells and (B) Karpas 299 cells. Cells were treated with/without sulfo-NHS-biotin, and incubated with 1F7 or IgG_1 _for (A) 0, 1 and 3 hours, or for (B) 1 hour. The cells were separated into nuclear and membrane fractions, immunoprecipitated with CD26 mAb, and immunoblotted. (C) J/Wt cells and J/CD26 cells were treated with biotin-labeled antibodies (1F7 or control IgG_1_) and separated into nuclear and membrane fractions. The biotinylated antibody-bound proteins were purified on beads immobilized to NeutrAvidin and separated by SDS-PAGE. Representative CD26 expression was shown. Similar results were obtained in three independent experiments.

The observed enhanced localization of CD26 in the nucleus, and the concomitant decrease of CD26 in the membrane fraction, following 1F7 treatment, suggests that CD26 on the cell surface moves into the nucleus as a result of stimulation with 1F7. Thus, we biotin-labeled J/CD26 cell surface proteins and these were chased following 1F7 treatment. Biotin-labeled CD26 levels increased dramatically in the nuclear fraction within one hour following 1F7 treatment (Fig. 4A). In contrast with the nuclear fraction, biotin-labeled CD26 decreased in the membrane fraction within one hour following 1F7 treatment (Fig. 4A). Thus, cell surface CD26 appears to translocate into the nucleus following 1F7 treatment. In addition, it appeared that this translocation of CD26 into the nucleus from the cell surface was constant as biotin-CD26 was always observed in the nuclear fraction, before and after treatment with IgG_1 _(Fig. 4A). Karpas 299 cells express CD26 endogenously, and our previous report has shown that cell cycle arrest in Karpas 299 cells related to p21 expression is induced following 1F7 treatment [6]. Therefore we investigated the effect of 1F7 on translocation of CD26 into nucleus of Karpas 299 cells. As a result, like J/CD26 cells, biotin-CD26 increased in the nuclear fraction of Karpas 299 cells following stimulation with 1F7, compared with IgG_1 _(Fig. 4A and 4B). These results indicate that cell surface CD26 is constantly translocated into the nucleus, and 1F7 treatment enhances this translocation. On the other hand, in JMN cells, these effects of 1F7 were slight. We think this discrepancy may be explained by the difference of cell lineage and nuclear CD26 amount before the 1F7 treatment (Fig. 1A and 1B).

To confirm the augmentation of CD26 translocation into the nucleus by 1F7, we assessed whether biotin-labeled 1F7 could enhance this CD26 translocation in J/Wt and J/CD26 cells. CD26 bound to biotin-1F7 was detected in the nuclear fraction and the membrane fraction of J/CD26 cells, in contrast with biotin-IgG_1 _(Fig. 4C, top panel). The biotin-1F7 enhanced CD26 translocation into the nuclear fraction from the membrane fraction (Fig. 4C, middle panel), confirming that the biotinylation of 1F7 did not influence the induction of CD26 translocation. These data show that the specific binding of 1F7 to a particular epitope on CD26 may induce the translocation of cell surface CD26 into the nucleus. However, the mechanism of this translocation of CD26 into nucleus, and the augmentation by 1F7 treatment, remains unknown.

Previous reports show that membrane-associated proteins, such as the epidermal growth factor receptor (EGFR) family [12-14], fibroblast growth factor receptor (FGFR)-1 [15,16], heparin-binding EGF-like growth factor (HB-EGF) [17], and CD40 [18], are shuttled from the plasma membrane into the nucleus. The translocation of these molecules into the nucleus can also be elevated by stimulation with, for example, specific ligands for the receptor, and the ectodomain shedding of proHB-HGF at the extracellular region on the plasma membrane. These membrane-associated molecules transmit specific signals from the cell surface to the nucleus after stimulation, and travel directly to the nucleus via internalization. The receptors that are transported into the nucleus have a common function as transcription regulators. There is no evidence that CD26 functions as transcriptional regulator, but a study which silenced CD26 using small interfering RNA showed an increase in basic FGF in prostate cancer cells *in vitro *[19]. Altered gene expression, mediated by CD26, may indicate a novel function of CD26 in the nucleus. DPPIV activity in the nucleus may be also implicated in the intra-nucleus dynamics by regulating the activation of unknown substrates of DPPIV in the nucleus.

These results permit us to speculate that nuclear events, such as transcription or cell growth, may be regulated by nuclear CD26.

## Conclusion

We observed that CD26 was constantly present in the nucleus of CD26+ cancer cells and the nuclear translocation of CD26 from cell surface was augmented by the treatment with anti-CD26 monoclonal antibody with anti-tumor effect in epitope-specific manner. These results suggest that intra-nuclear CD26/DPPIV may play an important role in the proliferation or survival of cancer cells. The CD26 therefore represents a potential new therapeutic target in human cancer could be considered.

## Methods

### Cell culture

Karpas 299 cells (derived from an anaplastic, large T-cell lymphoma) [6], JMN cells (derived from a mesothelioma) [4], Jurkat cells (J/Wt), and Jurkat cells containing a CD26 expression vector (J/CD26) [8], were cultured at 37°C in RPMI 1640 medium supplemented with 10% fetal bovine serum, 100 units/mL penicillin, and 100 μg/mL streptomycin.

### Subcellular fractionation and immunoblotting

Cells were incubated in medium with/without the CD26 mAbs, 1F7 or 5F8 (2 μg/mL), or control mouse IgG_1 _(Dako Cytomation, Denmark). After appropriate incubation, cells were harvested and washed with PBS. Membrane, cytoplasm and nuclear fractions were extracted using the Qproteome cell compartment kit, and according to the manufacturer's instructions (Qiagen, Hilden, Germany). The membrane fraction contains endosomes and membrane-compartmented organelles, such as mitochondria and the ER, as well as cell surface plasma membrane. Protein concentrations of each fraction were determined using the BCA protein assay kit (Pierce Biotechnology, Rockford, IL, USA). Equal amounts of protein were subjected to 10% SDS-PAGE and transferred to a polyvinylidene fluoride (PVDF) membrane. The membranes were probed with the following antibodies: anti-CD26 polyclonal antibody (pAb) (R&D systems, Mineapolis, MN, USA), anti-Na^+^/K^+^ATPase mAb (Santa Cruz Biotechnology, Inc., Santa Cruz, CA, USA) as a plasma membrane marker, anti-culreticulin mAb (BD Pharmingen, San Diego, CA) as an ER marker, anti-calpain-1/2 mAb (Calbiochem, La Jolla, CA) as a cytoplasmic marker, anti-lamin A/C mAb (Santa Cruz) as a nuclear marker, and anti-nucleostemin Ab (Qiagen) as a nuclear marker. Signals were revealed by enhanced chemiluminescence (ECL). Relative CD26 amounts for representative experiment were quantitatively analyzed with the Image Quant 350 software (GE healthcare, UK) and represented as percentage of CD26 expression in each fraction.

### Immunoelectron microscopic analysis

Karpas 299 cells and JMN cells were fixed on ice overnight in 0.1 M cacodylate buffer (0.1% glutaraldehyde and 4% paraformaldehyde, pH 7.4). The cells were dehydrated by two, 5-min incubations in 50, 70, 95, and 100% dimethylformamide in water. Cell pellets were incubated in dimethylformamide/lowicryl (1:1) for 30 min at room temperature. Sections (8 nm) were cut and mounted on copper mesh with 150 grids, incubated with a CD26 pAb (Santa Cruz) overnight, washed four times with PBS, and labeled for 60 min with a secondary, 15-nm gold-immunolabeled, anti-rabbit antibody. Sections were washed with 2% uranyl acetate, followed by 4% lead citrate and visualized with an electron microscope.

### DPPIV activity assay

Nuclear and cytosol fractions were prepared as described [15,20], with some modifications. Briefly, cells ware washed with PBS and resuspended in 400 μL of ice-cold buffer containing 10 mM HEPES (pH 7.4), 10 mM KCl, 0.1 mM EDTA, 0.1 mM EGTA, and protease inhibitor (Roche, Mannheim, Germany). The cells were allowed to swell on ice for 15 min, at which time 25 μL of 10% NP-40 was added, and the cells vortexed vigorously for 10 s. Nuclei were pelleted by centrifugation, washed twice with ice-cold buffer containing NP-40, and resuspended in 150 μL nuclear extract buffer containing 20 mM HEPES (pH 7.4), 0.4 M NaCl, 1 mM EDTA, 1 mM EGTA and protease inhibitor (Roche). Equal amounts (5 μg) of fractionated proteins were seeded onto 96-well plates and incubated in the presence or absence of diprotin A (an inhibitor of the DPPIV enzyme) (200 μM; Sigma, St. Louis, MI, USA), and the DPPIV substrate Gly-Pro-aminoluciferin (DPPIV-Glo protease assay; Promega, Madison, WI), and gently mixed. Plates were incubated for 30 min in the buffer system optimized for DPPIV and luciferase activity. Luciferase activity was assessed with a luminometer.

### Cell surface biotinylation and Immunoprecipitation

Cells were washed twice with PBS containing 1 mM CaCl_2 _and 0.5 mM MgCl_2 _(PBS-CM), at 4°C, and incubated for 12 min at 4°C in 1.0 mg/mL N-hydroxysulfosuccinimide (sulfo-NHS)-Biotin (Pierce) dissolved in PBS-CM. To quench unreacted biotin, cells were washed three times with PBS-CM plus 50 mM glycine and washed twice with PBS [21,22]. After incubation with the indicated antibodies, immunoprecipitation was performed using the CD26 mAb, 1F7 (2 μg), with equal amounts of protein from each fraction, overnight at 4°C. The antibody was bound directly to Protein A Sepharose beads for 1 h at 4°C. The beads were washed four times with NET-2 buffer [23] and the bound proteins eluted by directly adding SDS loading buffer to the beads.

### Biotinylation of antibodies

1F7 and control mouse IgG_1 _were biotinylated using a biotin labeling kit (Roche). Cells were incubated with culture medium containing the biotin-labeled antibodies for 1 h at 37°C. After washing with PBS, the cells were subcellular fractionated. Equal amounts of protein were precipitated with immobilized NeutrAvidin (Pierce) overnight at 4°C. The beads were washed four times with NET-2 buffer, and precipitated, biotinylated proteins were eluted by adding SDS sampling buffer.

## Abbreviations

mAb: monoclonal antibody; pAb: polyclonal antibody; DPPIV: dipeptidyl peptidase IV; ADA: adenosine deaminase; ECM: extracellular matrix; PBS: phosphate-buffered saline; BCA: bicinchoninic acid; SDS-PAGE: sodium dodecyl sulfate polyacrylamide gel electrophoresis; ER: endoplasmic reticulum; sulfo-NHS: N-hydroxysulfosuccinimide

## Competing interests

The authors declare that they have no competing interests.

## Authors' contributions

KY: Experimental design, interpretation of data and all experiments. MH: Experiments (measurement of enzyme activity). DW: Experiments (cell culture and immunoblotting). KO: Experiments (analysis with antibody). MS: Experiments (immuno-electron microscopy). CM: Experiments (production of antibody). TY: Experimental design, conception and manuscript preparation. All authors have read and approved the final manuscript.
